# Performance of penicillinase detection tests in *Staphylococcus epidermidis*: comparison of different phenotypic methods

**DOI:** 10.1186/s12866-020-01929-x

**Published:** 2020-08-05

**Authors:** Benjamin Aubry, Carole Lemarié, Rachel Chenouard, Marie Kempf, Matthieu Eveillard, Hélène Pailhoriès

**Affiliations:** 1 Laboratoire de bactériologie, CHU Angers, 4 rue Larrey, 49933 Angers, France; 2 CRCINA, Inserm, Université de Nantes, Université d’Angers, 44200 Angers, Nantes, France; 3 Laboratoire HIFIH, UPRES EA3859, SFR 4208, Université d’Angers, Angers, France

**Keywords:** Penicillinase detection, *Staphylococcus epidermidis*, Disk diffusion method, Zone edge test, Nitrocefin test, MIC

## Abstract

**Background:**

*Staphylococcus epidermidis* is the leading coagulase negative staphylococci (CoNS) species associated with healthcare associated infections. In order to de-escalate antimicrobial therapy, isolates of *S. epidermidis* lacking the *bla*Z gene should be eligible for targeted antimicrobial therapy. However, testing the susceptibility of coagulase negative staphylococci (CoNS) to penicillin G is no longer recommended by EUCAST, given the low performances for penicillinase detection in CoNS. The objective of this work was to determine a phenotypic method with high performance for detecting penicillinase production in *S. epidermidis*.

**Results:**

Four techniques for the detection of penicillinase production (disk diffusion, zone edge test, nitrocefin test, Minimal Inhibitory Concentration (MIC) by automated system Vitek2®) were evaluated on 182 *S. epidermidis* isolates, using identification of *bla*Z gene by PCR as the reference method. The performance of the methods for penicillinase detection was compared by the sensitivity, the specificity, the negative predictive value and the positive predictive value, and with Cohen’s kappa statistical test. Among the 182 *S. epidermidis* included in this study, 55 carried the *blaZ* gene. The nitrocefin test, characterized by a poor sensitivity (91%), was therefore excluded from *S. epidermidis* penicillinase detection. The algorithm proposed here for the penicillinase detection in *S. epidermidis* involved two common antimicrobial susceptibility techniques: disk diffusion method and MIC by Vitek2® system. Disk diffusion method, interpreted with a 26 mm breakpoint for penicillin G, was associated with a high sensitivity (98%) and specificity (100%). This method was completed with zone edge test for *S. epidermidis* with penicillin G diameter from 26 to 35 mm (sensitivity of 98%). The Vitek2® system is associated with a low sensitivity (93%) and a high specificity (99%) This low sensitivity is associated with false negative results, in isolates with 0.12 mg/L Penicillin G MIC values and *blaZ* positive. Thus for penicillin G MIC of 0.06 mg/L or 0.12 mg/L, a second step with disc diffusion method is suggested.

**Conclusions:**

According to our results, the strategy proposed here allows the interpretation of penicillin G susceptibility in *S. epidermidis* isolates, with an efficient detection of penicillin G resistance.

## Background

*Staphylococcus epidermidis* is the first coagulase-negative *Staphylococcus* (CoNS) documented in the early twentieth century, with a taxonomic description in 1908 as *Albococcus albus* [1]. This one is also one of the most frequent CoNS isolated in humans [1]. Indeed, among skin CoNS, *S. epidermidis* is the most common species colonizing the body surface [2–4]. However, this bacterium is also responsible for healthcare-associated infections (HAI) [5–7]. Indeed, *S. epidermidis* has been reported as the leading bacterial species associated with device-associated HAI, such as catheters or prosthetic cardiac valves [1, 8, 9]. Native valve endocarditis, infections in neonates and bacteremia in neutropenic patients have also been described [1].

The first resistance of *S. epidermidis* to penicillin has been firstly detected, in four fatal cases of subacute bacterial endocarditis in 1949 [10]. Today, more than 90% of *S. epidermidis* are considered as resistant to penicillin [11, 12]. This resistance affects penicillins V, G and A, ureidopenicillins and carboxypenicillins. The susceptibility of *S. epidermidis* to these molecules is restored by the addition of beta-lactamase inhibitors such as clavulanic acid. In addition, many other classes of the beta-lactam family remain sensitive (penicillin M, cephalosporins, carbapenems). A major mechanism of penicillin resistance is penicillinase production, an enzyme that hydrolyzes the beta-lactam ring of penicillin [13, 14]. This plasmid-mediated beta-lactamase is encoded by the *bla*Z gene [15]. The *blaZ* gene is associated with a repressor gene *blaI*, and a signal transducer-sensor protein *blaR1* [15]. The *blaZ* gene expression is upregulated following the interaction between blaR1 and beta-lactams, which is responsible for an inactivation of the repressor blaI. The *mecA* gene can also be mentioned as another gene responsible for penicillin resistance but also for resistance to beta-lactams in general in *S. epidermidis*.

The inducible mechanism of penicillinase production in presence of beta-lactams makes it difficult to identify in CoNS, and thus to conclude on the penicillin resistance. No European recommendations prevail for penicillin susceptibility testing in *S. epidermidis*, EUCAST stating that no currently available method is reliable for penicillinase production detection in CoNS. All strains must de facto be reported as resistant to benzylpenicillin, ampicillin, amoxicillin, piperacillin and ticarcillin. However, for *S. aureus,* EUCAST recommends the use of disk diffusion method, zone diameter measure and zone edge inspection being more reliable than Minimal Inhibitory Concentration (MIC) determination for the detection of penicillinase [16]. Chromogenic tests are also available for the penicillinase detection, although they are not recommended for this use. Nevertheless, penicillins are of considerable interest because these molecules have few side effects, significant bactericidal properties, a narrow spectrum and low therapeutic costs [14].

The aim of this study was to compare different methods for the diagnosis of penicillinase production and to determine a strategy based on phenotypic methods for asserting the production of penicillinase in *S. epidermidis*.

## Results

### Bacterial isolates

In this study, 182 non-duplicate methicillin-susceptible *S. epidermidis* clinical isolates (MSSE) were included. Among them, 127 strains interpreted susceptible to penicillin G and 55 strains resistant to this antibiotic were included [14]. *S. epidermidis* were mainly isolated from blood cultures (59), urines (44), tissues (14), liquids (20), catheters (20), implantable venous access devices (9), CSF (5) and other superficial samples (11).

### blaZ gene detection

A total of 127/182 isolates (69.8%) were found *bla*Z negative while 55/182 isolates (30.2%) carried the *bla*Z gene.

### Diffusion method - reading inhibition diameters

**Fig. 1:**
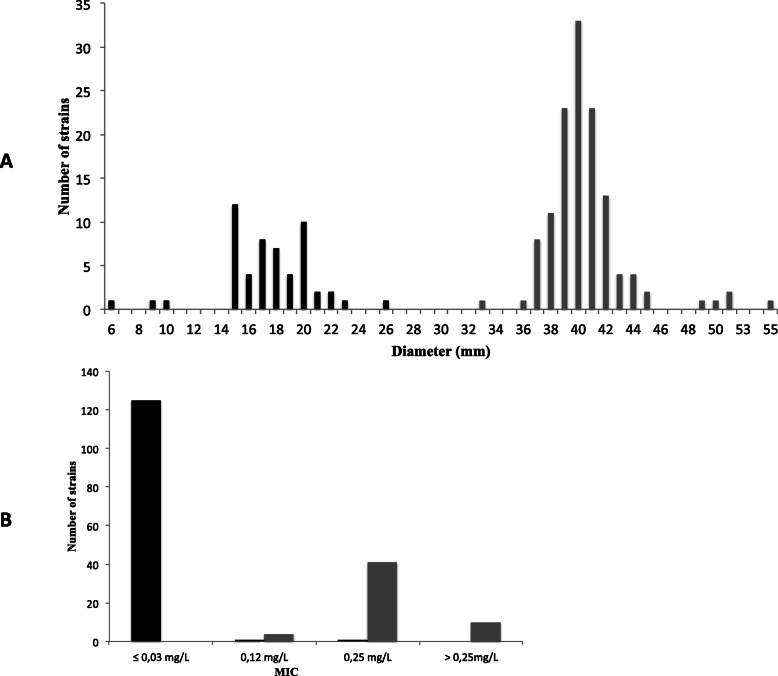
**a** Dispersal of diffusion diameters according to the presence of the *bla*Z gene. **b** Distribution of Penicillin G MIC (Vitek 2®) for 182 *S. epidermidis* isolates according to the presence of *bla*Z gene. Black bars represent *blaZ* positive *S. epidermidis* isolates and grey bars represent *blaZ* negative *S. epidermidis* isolates

Considering the 182 isolates, penicillin G inhibition zone diameters ranged from 6 to 55 mm. The 55 *bla*Z positive *S. epidermidis* isolates displayed diameters between 6 and 26 mm. The 127 *bla*Z negative isolates formed diameters between 33 and 55 mm (Fig. 1a).

Using the cut-off value of 26 mm (EUCAST recommendations for *S. aureus* [16]), the sensitivity of the method was 98%, the specificity of 100%, the negative predictive (NPV) value of 99% and the positive predictive value (PPV) for the detection of *blaZ* positive isolates was 100% (Table 1). One *S. epidermidis* isolate with a diameter of 26 mm, thus interpreted as susceptible to penicillin G following the guidelines previously mentioned, was in fact *blaZ* positive. A very strong agreement was observed between penicillin G zone diameters and *blaZ* PCR (κ = 0.987).

**Table 1:** Performances of Penicillin G zone diameter, nitrocefin disk test, Zone edge test and Penicillin G MIC determination (Vitek 2®) for the detection of penicillinase production in 182 *S. epidermidis* isolates and comparison of zone edge test results between the 5 investigators

Phenotypic method for penicillinase detection	TP (N)	FP (N)	TN (N)	FN (N)	Total (N)	Se (%)	Sp (%)	PPV (%)	NPV (%)
Diameter	54	0	127	1	182	98	100	100	99
Nitrocefin	50	0	127	5	182	91	100	100	96
Zone edge test	54	20	107	1	182	98	84	73	99
MIC	51	1	126	4	182	93	99	98	97
zone edge test reading/investigators									
Investigator 1	54	21	106	1	182	98	83	72	99
Investigator 2	54	19	108	1	182	98	85	74	99
Investigator 3	53	43	84	2	182	96	66	55	98
Investigator 4	55	28	99	0	182	100	78	66	100
Investigator 5	54	5	122	1	182	98	96	93	99
Final result	54	20	107	1	182	98	84	73	99

*blaZ* PCR was used as the reference method

*TP* true positive, *FP* false positive, *TN* true negative, *FN* false negative, *Se* Sensitivity, *Sp* Specificity, *PPV* Positive Predictive Value, *NPV* Negative Predictive Value, *(N)* Number of isolates

### Diffusion method - nitrocefin disk test

The 127 *blaZ* negative *S. epidermidis* isolates had a negative nitrocefin disk test result (unchanged or yellow colour after 60 min). Among the 55 *blaZ* positive isolates, 50 had a positive result, but 5 had a negative nitrocefin test. These 5 false negative (FN) results were associated with penicillin G inhibition zone diameters ranging from 16 to 20 mm. The nitrocefin disk test had a sensitivity of 91%, a specificity of 100%, a positive predictive value of 100% and a negative predictive value of 96% (Table 1). A very strong agreement was observed between nitrocefin disk test and *blaZ* PCR (κ = 0.933).

### Zone edge test

To evaluate the performance of this method, we first evaluated the sensitivity and specificity of the zone edge test interpretation of each investigator. Then, the interpretation of all the investigators were combined, selecting the result of the majority of the investigators for each *S. epidermidis* isolate (at least 3 interpretations within the 5) (Table 1). The 5 investigators selected for zone edge test reading were skilled bacteriologists with a 2 to 25-year experience.

Sensitivity differed slightly between the five investigators, with a sensitivity greater than or equal to 96% for all readers (from 96 to 100%). On the contrary, a wide variation of the specificity was observed, ranging from 66 to 96% (Table 1). No correlation could be established between the length of the laboratory practice and the performances of zone edge test reading. Combining the results of the five investigators, zone edge test had a sensitivity of 98%, a specificity of 84%, a positive predictive value of 73% and a negative predictive value of 99% (Table 1). Indeed, on the 127 *blaZ* negative *S. epidermidis* isolates, 20 were interpreted with a positive zone edge test, and on the 55 *blaZ* positive *S. epidermidis* isolates, one was interpreted with a negative zone edge test.). A strong agreement was observed between nitrocefin disk test and *blaZ* PCR (κ = 0.751).

### Penicillin G MIC – Vitek 2® method

A sensitivity of 93% and a specificity of 99% were assessed for this method, with a negative predictive value of 97%, and a positive predictive value of 98% (Table 1). Of the 127 isolates lacking the *bla*Z gene, 125 corresponded to MICs ≤0.03 mg/L, one had a MIC of 0.12 mg/L, and one had a MIC of 0.25 mgL, and was thus considered as a false positive. On the 55 *bla*Z positive isolates, 4 had a MIC of 0.12 mg/L and considered as false negative results, 41 had a MIC of 0.25 mg/L, and 10 with a MIC result > 0.25 mg/L (Fig. 1b). A very strong agreement was observed between nitrocefin disk test and *blaZ* PCR (κ = 0.934).

## Discussion

The aim of this work was to determine a sensitive and specific phenotypic method for the diagnosis of *S. epidermidis* penicillinase production. Here, *blaZ* PCR has been used as the reference technique for the detection of penicillinase. The objective of this study was to assess the performance of different phenotypic methods for the diagnosis of penicillinase production.

The disk diffusion method as used in this study yielded 98% sensitivity and 100% specificity results for the detection of *S. epidermidis* penicillinase production. Few studies exist on the performance of penicillinase detection in CoNS. A study of Kaase et al. also assessed the performance of disk diffusion method for the penicillinase detection, but only on *Staphylococcus aureus* isolates. In this last work, disk diffusion method was interpreted following CLSI recommendations, using a 10 unit penicillin G disk and interpreted with a zone diameter breakpoint of 28 mm (diameters ≤28 mm interpreted as resistant to penicillin). A sensitivity of 57% was obtained for the detection of penicillinase with this method on 197 isolates of *S. aureus* [17]. Also using CLSI recommendations, Ferreira et al. achieved 72% sensitivity results in 101 isolates of *Staphylococcus* (including 17% of *S. aureus* and 73% of CoNS isolates) [18]. The sensitivity results obtained in these studies seem therefore much lower than the result obtained here for *S. epidermidis* isolates. According to the EUCAST recommendations, the susceptibility to penicillin G is determined by using a 1 unit penicillin G disk and an inhibition zone diameter of 26 mm. In a recent study [19], Papanicolas et al. analyzed the production of penicillinase by disk diffusion method on 157 strains of *S. aureus* by comparing the European and American recommendations. For these strains, the sensitivity and specificity according to the EUCAST recommendations were respectively of 100 and 99%, as compared to 66 and 100% with CLSI recommendations. Thus, this last study supports the fact that EUCAST recommendations are a better predictor of beta-lactamase production. This should be taken into account for the analysis of performance of this method for penicillinase detection in *S. epidermidis*. By using EUCAST recommendations for *S. aureus*, good performances were obtained for the detection of *S. epidermidis* penicillinase in our study. The absence of the *bla*Z gene in the 127 strains with a diameter greater or equal to 33 mm supports the specificity of this test, and highlights the relability to conclude to the susceptibility to penicillin G beyond this diameter. However, the number of *S. epidermidis* isolates with a diameter between 26 and 35 mm in this study seems insufficient to exclude the presence of *blaZ* in isolates ranging in these values of penicillin G diameter, especially given the fact that a false negative result has been observed for a *blaZ* positive *S. epidermidis* with a 26 mm penicillin G diameter. For this last isolate, zone edge test was positive for the 5 investigators (sharp zone edge), with a positive nitrocefin and a MIC value > 0.25 mg/L. Thus, other diagnosis method should be used for the detection of penicillinase production in *S. epidermidis* isolates ranging from 26 to 35 mm.

A sensitivity of 91% and a specificity of 100% were obtained for the nitrocefin test. The performances of this test for the detection of *Staphylococcus* penicillinase vary widely in the literature. For *S. aureus* isolates, sensitivity results range from 39 to 92% [17, 19, 20]. Ferreira et al. also analyzed the performances of this method on different species of *Staphylococci*, and conclude with a sensitivity of 29%, which is much lower than the performance results obtained in this study [18]. However, we confirm here the results of other reports, outlining a lower sensitivity of nitrocefin test comparing with other phenotypic tests, including zone edge test [18–20]. The great variability of these results makes it difficult to consider this technique as reliable for the detection of beta-lactamase production in *Staphylococcus*. The performance variation according to studies could also be associated with the different reagents used for the realization of this technique (nitrocefin solution or disks), and to the localization of colonies picked for the test (on penicillin G zone edge as in this study, on cefoxitin zone edge or without any induction). Here, this test, associated with the lowest negative predictive value within all the phenotypic methods tested (96%), should not be recommended for the detection of penicillinase in *S. epidermidis*.

The results of the inspection of the zone edge are contrasted. EUCAST recommends the inspection of penicillin G zone edge in *S. aureus*, for isolates with diameters ≥26 mm. Here, we applied this method to all the *S. epidermidis* isolates. The sensitivity is 98%, but the low specificity result (84%) seems insufficient to use this method. Indeed, the positive predictive value, associated with the ability of this technique to correctly assign isolates as *blaZ* positive, is only of 73%. The good sensitivity recorded in our study for this test is supported by the literature. Indeed, a sensitivity greater than 90% was obtained by testing different species of *Staphylococci* [18], and a sensitivity of 100% was recorded by testing different *S. aureus* isolates [19]. In a study of Gill et al. carried out on 260 strains of *S. epidermidis*, the sensitivity determined for this method was 95%. However, in this same work, the appearance of the zone edge was uninterpretable for 17 *S. epidermidis* (14 penicillinase producers and 3 non-producers) [21]. This study highlights the difficulties encountered in the interpretation of this test on some *S. epidermidis* isolates. On the other hand, the subjective aspect of the method (associated with human interpretation) can explain the variability of the results and the low specificity obtained for this test. In a study of Hombach et al. performed on *S. aureus* isolates, a wide variation in the results of the zone edge test have been observed depending on the level of experience of the investigator. Similarly, the results of the zone edge test varied between the different readers in our study If the sensitivity varied slightly (from 96 to 100%), the specificity could vary by 30% between two investigators. Discrepancies between the microbiologists in the interpretation of the zone edge test were noted for approximately one third of *S. epidermidis* isolates (58/182), with a total of 111 interpretation errors. For 56 of these 58 discordant strains, the penicillin G diameter was greater or equal to 33 mm. Thus, an error has been made by at least one of the microbiologists, on 44% of the 127 *bla*Z negative isolates. These errors seemed therefore relatively recurring between the different investigators. It is interesting to note, however, that for the only *S. epidermidis* isolate with a discordant result by disk diffusion method (diameter ≥ 26 mm and *bla*Z positive), the zone edge test interpretation was concordant for all microbiologists. Furthermore, combining the results of all investigators, an error occurred in 11,5% of the *S. epidermidis* isolates mainly consisting in false positive interpretations (20 errors on 21). This phenotypic method therefore should appear as an additional criterion for strains with a penicillin G diameter between 26 and 35 mm. However, the difficulty of zone edge interpretation associated with the subjective nature of this test should be duly noted, so as the risk to slightly overestimate the number of *S. epidermidis* penicillinase producer.

The results of the microdilution method, carried out here with the Vitek2® system, were associated with a sensitivity of 93% and a specificity of 98%. In addition, in our study, and as reported in the literature for *S. aureus* isolates, no strain with MIC values ≤0.03 mg/L carried the *bla*Z gene [17, 20, 22]. In our study, no strain had a 0.06 mg/L MIC, 4 out of 5 strains with a MIC of 0.12 mg/L and 40 strains out of 41 with a MIC of 0.25 mg/L were *bla*Z positive, attesting to the low reliability of this method to detect penicillinase production for MICs between 0.06 and 0.12 mg/L. In a study of Richter et al. including 448 *S. aureus* strains, 96% of strains with MIC = 0.06 mg/L and 68% of the strains with MIC = 0.12 mg/L were *bla*Z negative. The results of the study conducted by Kaase et al. with 197 *S. aureus* strains were similar, 94% of strains with a MIC = 0.06 mg/L and 77% of strains with a MIC = 0.12 mg/L, being *bla*Z negative. Thus, on the results recorded with strains of *S. aureus* do not seem comparable to those obtained in the present study, with isolates of *S. epidermidis*. These results prompt us in the context of the study of *S. epidermidis* to establish a breakpoint of this method to 0.03 mg/L to attest the absence of beta-lactamase, and continue testing strains with MIC from 0.06 to 0.12 mg/L with another technique to ensure the absence of production of penicillinase. For laboratories that only have an antimicrobial susceptibility testing system by the microdilution method, these results are important for establishing a decision algorithm for the detection of penicillinase production.

One limitation that could be pointed in this study is using *blaZ* PCR as the reference method. Indeed, the transcription level of *blaZ* has not been investigated. Indeed a correlation could maybe have been made between the level of transcription of the *blaZ* gene and the false positive results obtained with some phenotypic methods. Such investigations should be interesting to perform in further studies.

**Fig. 2:**
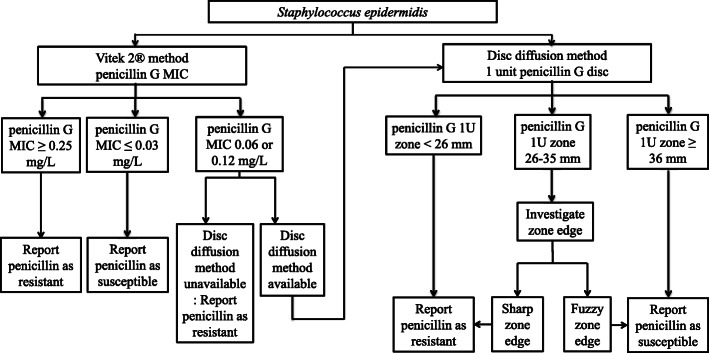
Suggested diagnosis approach by disk diffusion method or Vitek 2® method to determine susceptibility of *S. epidermidis* to penicillin G

In summary two methods seem particularly interesting to determine the susceptibility of *S. epidermidis* to penicillin G, the disk diffusion method and MIC Vitek 2® method. Both methods are available in most bacteriology laboratories, and seem here to have good performance for the detection of penicillinase production in *S. epidermidis*, but using revised breakpoints. The nitrocefin test is associated with a poor sensitivity. Concurrently, the inspection of the zone edge could play a role in complement of the diameter, especially for isolates with a 26 to 35 mm diameter, taking into account the overestimation of penicillinase producers with this technique which could avoid some false negative results. This approach is summarized in Fig. 2. For laboratories performing antibiograms by microdilution, the diagnosis approach is also suggested in Fig. 2.

## Conclusions

We provide here an original diagnosis strategy, based on phenotypic methods, for the determination of *S. epidermidis* susceptibility to Penicillin G. The diagnosis approach proposed here is valid for MSSE isolates, so only after the inspection of the methicillin susceptibility of the *S. epidermidis* isolates. Indeed, in order to de-escalate antimicrobial therapy, MSSE isolates lacking the *bla*Z gene should be eligible for targeted antimicrobial therapy. The diagnosis approach suggested here should allow the interpretation of Penicillin G susceptibility in *S. epidermidis* isolates.

## Methods

### Bacterial isolates

In this study, 182 non-duplicate methicillin-susceptible *S. epidermidis* clinical isolates (MSSE), isolated in the laboratory of bacteriology of a French teaching hospital between January 2015 and August 2018, were included. *S. epidermidis* strains interpreted susceptible to penicillin G – following 2015 to 2017 CA-SFM recommendations (Committee for antibiotic susceptibility testing of the French Society of Microbiology) - were selected [14]. The penicillin G susceptibility of *S. epidermidis* isolates was evaluated by the determination of the MIC of penicillin G by Vitek® 2 method (bioMérieux, Marcy l’Etoile, France) and on the measurement of inhibition zone diameter of penicillin G by disk diffusion method. In addition, *S. epidermidis* strains resistant to penicillin G, excluding methicillin-resistant *S. epidermidis*, were also included.

### Bacterial cultures

The *S. epidermidis* isolates were subcultured from Stock Culture storage medium (Bio-Rad®, Hercules, USA) on Columbia sheep blood agar (Oxoid®, Dardilly, France) and incubated 24 h at 35 +/− 2 °C. Identification of bacterial colonies was verified by Matrix Assisted Laser Desorption Ionization - Time of Flight mass spectrometry (MALDI-TOF MS) (bioMérieux®, Marcy l’Etoile, France).

### blaZ gene detection

The DNA was extracted from bacterial colonies using a lysostaphin-based extraction protocol. Genetic detection of *blaZ* genes was performed by using an in-house real-time PCR, amplifying a 165 bp fragment of the *blaZ* gene (nucleotide position 665–830). The total DNA extracted from *S. epidermidis* colonies was amplified by using *bla*Z –for primer (5′-TGCTGATAAAAGTGGTCAAGCA-3 ‘), *bla*Z-rev primer (5’-ACACTCTTGGCGGTTTCACT-3′), *blaZ*-dye probe (5′-FAM-TCCTAAGGGCCAATCTGAACCTATTGT-BHQ1–3′) (Eurofins®), Brilliant III ultrafact QPCR Low ROX Master Mix (Agilent®) and water for PCR (Invitrogen®) for a final volume of 20 μL for each reaction mix. The PCR protocol was performed on MX 3000 (Agilent®) and included an initial denaturation at 95 °C for 3 min followed by 45 denaturation cycles (95 °C for 15 s) and hybridization / elongation cycles (55 °C for 25 s). Some *blaZ* positive and negative *S. epidermidis* strains were used as controls for each PCR run (clinical *S. epidermidis* isolated in the laboratory previously proved as *blaZ* negative and positive, and respectively producer and non-producer of penicillinase).

### Diffusion method - reading inhibition diameters

The disk diffusion method was performed on Mueller-Hinton agar plates (Oxoid®, Dardilly, France) with a 0.5 Mac Farland bacterial suspension, and by using a 1 unit disk of penicillin G (Oxoid®, Dardilly, France) according to EUCAST recommendations [16]. The plates were incubated 20 +/− 4 h at a temperature of 35 +/− 2 °C. To determine the susceptibility to penicillin, a breakpoint value of 26 mm, following EUCAST recommendations for *S. aureus* species, was used [16].

### Diffusion method - nitrocefin disk test

Nitocefin disk test has been used for penicillinase detection since a long time in bacteriology laboratories on several bacterial species, including Staphylococcus. If this chromogenic based method is no longer recommended by EUCAST for Staphylococci, including *S. aureus*, the aim here was to state the performance of this method on *S. epidermidis* isolates. The nitrocefin disk test (Remel®, San Diego, USA) was used according to the recommendations of the manufacturer. Briefly, colonies picked from the penicillin G inhibition zone edge were applied on a moistened nitrocefin disk. The test was interpreted, according to the indications of the supplier, 60 min after the beginning of the test and checked for a colour change. A pink/red test has been rated as a positive result, and a yellow or colorless test has been rated as negative.

### Zone edge test

The appearance of the zone edge of penicillin G inhibition zone was also determined. Zone edge test is recommended by EUCAST for penicillinase detection in *S. aureus* isolates with a Penicillin G diameter greater than 26 mm. The aim here was to state the performance of this method in *S. epidermidis* isolates. Five experienced bacteriologists have inspected the appearance of the zone edges and classified as following: sharp if well defined (therefore resistant to penicillin G) and fuzzy if not clearly delimited (therefore susceptible to penicillin G). Each reading was performed independently without consultation between the investigators. For each *S. epidermidis* isolate, the results of the five readers have been compiled, and the most frequent interpretation (given by at least 3 investigators) has been chosen as the final result. Thus, the final result for each isolate (fuzzy or sharp) was the result given by the majority of the readers.

### Penicillin G MIC – Vitek 2® method

The MICs of penicillin G were initially tested by the microdilution method, with the Vitek2® AST-P631 system (Vitek2®, bioMérieux, Marcy l’Etoile, France), testing penicillin G concentrations ranging from ≤0.03 mg/L to > 0.25 mg/L (≤ 0.03 mg/L, 0.06 mg/L, 0.12 mg/L, 0.25 mg/L and > 0.25 mg/L). When a discrepancy was observed between the MIC value of penicillin G and the *bla*Z PCR result, the MIC was determined a second time by the same technique to confirm this result.

### Results interpretation and analysis

The *bla*Z gene PCR was considered as the gold standard to attest the presence of a penicillinase. Each method tested in this study was compared with this reference method. Each discrepant result with the reference method (*blaZ* PCR) has been repeated once for confirmation. The performance of the different methods for penicillinase diagnosis was compared by the sensitivity, the specificity, the negative predictive value and the positive predictive value and a diagnosis approach was suggested considering the performance of the different methods. Furthermore, staistical analyses were performed using Cohen’s kappa test (SPSS v15.0). Indeed, a kappa coefficient between 0.81 and 1 indicated a very strong agreement between the results of the test and the blaZ PCR reference test. A kappa coefficient between 0.61 and 0.8 indicated a strong agreement between the two results. Conversely, a kappa coefficient of < 0.61 meant that the two results were not sufficiently in agreement.

## Data Availability

The datasets used and/or analysed during the current study are available from the corresponding author on reasonable request.

## Abbreviations

CLSI: Clinical and Laboratory Standards Institute
CoNS: Coagulase negative staphylococci
EUCAST: European Committee on Antimicrobial Susceptibility Testing
FN: False negative
FP: False positive
HAI: Healthcare-associated infections
MIC: Minimal inhibitory concentration
MSSE: Methicillin-susceptible *Staphylococcus epidermidis*
NPV: Negative predictive value
PCR: Polymerase chain reaction
PPV: Positive predictive value
